# The Effect of Chemerin on Cardiac Parameters and Gene Expressions in Isolated Perfused Rat Heart

**DOI:** 10.4274/balkanmedj.2017.1787

**Published:** 2019-01-01

**Authors:** Özden Kutlay, Ziya Kaygısız, Bilgin Kaygısız

**Affiliations:** 1 Department of Physiology, Eskişehir Osmangazi University School of Medicine, Eskişehir, Turkey; 2 Department of Pharmacology, Eskişehir Osmangazi University School of Medicine, Eskişehir, Turkey

**Keywords:** Animal experiment, chemerin, cyclic guanosine monophosphate, endothelial nitric oxide synthase, heart contractility

## Abstract

**Background:**

Chemerin is a novel chemoattractant adipokine expressed in cardiovascular system, and its receptor has been detected in the epicardial adipose tissue.

**Aims:**

To determine the effects of chemerin on the cardiac parameters and gene expressions in the isolated perfused rat heart.

**Study Design:**

Animal experiment.

**Methods:**

The hearts were retrogradely perfused with Langendorff technique to measure the cardiac parameters. The experimental groups were acutely treated with 10, 100, and 1000 nM doses of chemerin. Another group was given 10 μM L-nitric oxide synthase inhibitor for 5 min before 1000 nM chemerin administration. The real-time polymerase chain reaction was performed for detecting the expression of target genes.

**Results:**

All doses of chemerin significantly decreased the left ventricular developed pressure (max 35.33 Δ%, p<0.001), and +dP/dt_max_ (max 31.3 Δ%, p<0.001), which are the indexes of cardiac contractile force. In addition, 1000 nM chemerin reduced the coronary flow (max 31 Δ%, p<0.001). N(W)-nitro-L-arginine methyl ester antagonized the negative inotropic effect of chemerin on contractility. Chemerin induced a 2.16-fold increase in endothelial nitric oxide synthase mRNA and increased the cyclic guanosine monophosphate levels (p<0.001) but decreased the PI3Kγ gene expression (1.8-fold, p<0.001). Furthermore, all doses of chemerin decreased the CaV1.2 gene expression (1.69-fold, p<0.001).

**Conclusion:**

Acute chemerin treatment induces a negative inotropic action with the involvement of nitric oxide pathway, CaV1.2, and PI3Kγ on isolated rat heart.

The adipose tissue not only serves as an energy storage but also acts as an endocrine tissue that releases various proteins called adipocytokines (1). These proteins exhibit endocrine, autocrine, and paracrine effects and are involved in various physiological and pathophysiological events, such as food intake, insulin sensitivity, immunity, and inflammation (2). Chemerin is a 16 kDa adipocytokine consisting of 137 amino acid sequences (3); its receptors are synthesized mainly in the liver, adipose tissue, and cardiovascular system (4). Chemerin acts as a crucial protein in terms of physiological responses, such as inflammation and insulin resistance, adipose differentiation, and regulation of immune response, maturation, and metabolism (5). Chemerin may regulate the cardiovascular functions and gene expressions. Studies have reported that chemerin is associated with obesity, metabolic syndrome, and diabetes (6,7). In addition, circulating chemerin concentrations has been positively correlated to many obesity-related parameters, including body mass index, blood pressure, serum lipid, insulin, and cholesterol concentration (8). Previous studies have also demonstrated that chemerin plays a role in various cardiovascular pathologies, including the development of hypertension, (3) progression of atherosclerotic lesions (9), and impaired heart function in patients with dilated cardiomyopathy (5). Furthermore, chemerin-9 (nonapeptide of the chemerin S^157^ isoform) induces contraction in a concentration-dependent manner in isolated rat aorta (10). Chemerin-9 also causes contraction of the human saphenous vein and resistance in arteries and elevates blood pressure in rats (11).

Evidence suggests that (3,4,5,6,7,8,9,10,11) chemerin may influence cardiovascular functions and the expressions of PI3K alpha (PI3Kα), PI3K gamma (PI3Kγ), beta adrenergic receptor 1 (β1-AR), β2-AR, endothelial nitric oxide synthase (eNOS), sarcolemmal L-type Ca^2+^ channel (CaV1.2) genes, and the tissue levels of cyclic adenosine monophosphate (cAMP) and cyclic guanosine monophosphate (cGMP). However, the actions of chemerin on cardiovascular functions and expression of these genes have not been studied in isolated perfused rat hearts. Thus, we aimed to investigate the possible effects of chemerin on the left ventricular developed pressure (LVDP), maximal rate of pressure development (+dP/dt_max_), heart rate, coronary flow, monophasic action potential amplitude (MAPamp), MAP duration at 90% repolarization (MAP_90_), the genes mentioned above, and cAMP and cGMP levels.

## MATERIALS AND METHODS

Female Sprague-Dawley rats (250-350 g) were fed with a standard rat diet and housed in cages under 12 h light/12 h dark at 20 °C-25 °C.

The procedures in the present study were conducted in accordance to the “Guide to the Care and Use of Experimental Animals” by the Canadian Council of Animal Care and after receiving the approval of the Institutional Animal Care and Use Committee (IACUC) in Eskişehir Osmangazi University (IACUC approval no. 385/2014).

### Chemicals

Chemerin-9 was obtained from Anaspec (Fremont, CA, USA).

### Experimental groups

Four experimental groups were studied. Group 1: The hearts were perfused with only Krebs Henseleit solution (mK-Hs) for 30 min (control group). Groups 2, 3, and 4: The hearts were perfused with mK-Hs containing 10, 100, and 1000 nM chemerin, respectively. Group 5: The hearts were perfused with mK-Hs containing 10 μM NO synthase inhibitor N(W)-nitro-L-arginine methyl ester (L-NAME) for 5 min before 1000 nM chemerin administration.

### Experimental protocol

The animals were anesthetized with intraperitoneal injection of sodium thiopental (50 mg/kg) and given heparin (1000 IU). After sufficient anesthesia administration, the chest of the rats was opened. The heart was rapidly removed and arrested in ice-cold mK-Hs and then transferred to the Langendorff apparatus while perfusing with mK-Hs. The retrograde perfusion was performed in the non-circulating Langendorff mode under a constant pressure (60 mmHg), whereas the pulmonary artery was incised for the complete coronary drainage of the ventricles. To measure the cardiac contractile force, this study used the method previously described by He and Downey (12). A water-filled latex balloon was set to achieve a stable left ventricular end-diastolic pressure of 8 mmHg and then inserted into the left ventricle via the mitral valve and connected to a pressure transducer. Data acquisition was performed with a data acquisition and analysis system (Isoheart Software, Germany) to determine the pressure measurements. LVDP and +dP/dt_max_ were monitored and used as the contractility indexes. The contact electrode technique was applied by using MAP electrodes (Ag/AgCl_2_) to measure the MAPamp and MAP_90_ recordings. The hearts were included from the study if normal sinus rhythm or LVDP, +dP/dt_max_, and heart rate after the first 30 min of perfusion were more than 60 mm Hg, 2800 mmHg s^-1^, and 200 beat/min, respectively.

### mRNA preparation and real-time quantitative polymerase chain reaction

Total RNA was extracted by using Tripure reagent (Roche Life Science, Mannheim, Germany) according to the instruction manual (Roche, Life Science). The mRNA levels of all genes were measured by real-time quantitative polymerase chain reaction in a LightCycler 480 I (Roche Applied Science, Mannheim, Germany). The primer sets were as follows: PI3Kα, NM_013005.1; PI3Kγ, NM_022213.1; β1-AR, NM_012701.1; β2-AR, NM_012492.2; eNOS, NM_021838.2; CaV1.2, NM_012517.2; beta actin, NM_031144.3 (TIB-Molbiol, Berlin, Germany). Beta-actin gene was used as a housekeeping gene, and each sample was run as duplicate together with the negative control. Relative gene expression was normalized and calculated by using the 2^-ΔΔCT^ method.

### Enzyme-linked immunosorbent assay

The frozen tissues (50-100 mg) were treated with phosphate-buffered saline at 0 °C and centrifuged for 20 min at 446-1006×g. After the supernatant was extracted, the cAMP and cGMP concentrations of the aqueous phase were measured with a commercial enzyme immunoassay (YH Biosearch, Shanghai, China).

### Statistical analysis

SPSS for Windows (version 13.0, SPSS Inc, Chicago, USA) was used to conduct the statistical analysis. The normality of data distribution was evaluated with Shapiro-Wilk test and Kolmogorov-Smirnov test with Lilliefor’s correction. Thereafter, one-way analysis of variance and Tukey’s honest significant difference multiple comparisons post hoc test were used for the data analysis. The findings were reported as the mean ± standard deviation, and p values were considered statistically significant at less than 0.05.

## RESULTS

### Effects of chemerin on cardiovascular variables

**Figure 1:**
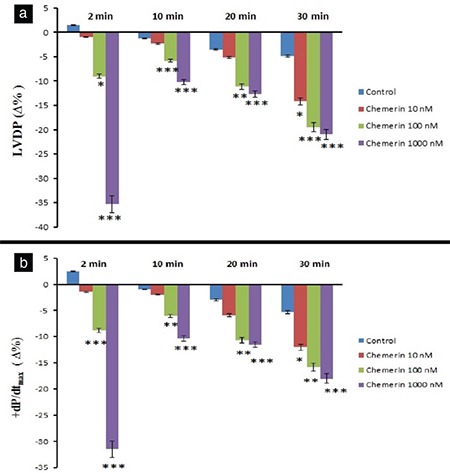
Time-dependent effect of chemerin on the LVDP (a) and +dP/dtmax (b). Δ% refers to the change as a percentage of the 0 min value, that is, the value obtained prior to chemerin administration in chemerin-treated groups and the value obtained after a 30 min stabilization period in the control groups. -Δ% denotes the decrease. *p<0.05, **p<0.01, and ***p<0.001 significantly different from the respective controls. LVDP: left ventricular developed pressure

**Figure 2:**
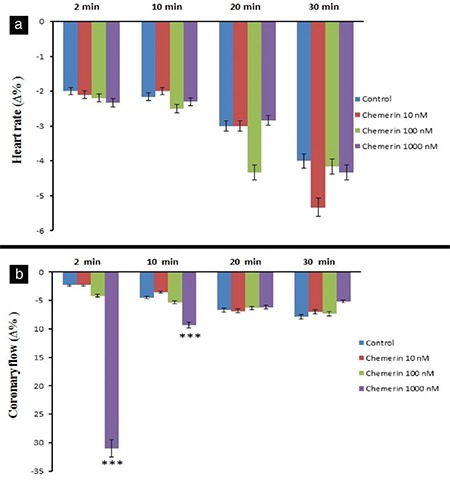
Time-dependent effect of chemerin on the heart rate (a) and coronary flow (b). Δ% indicates the change as a percentage of the 0 min value, that is, the value obtained prior to the administration of chemerin in the chemerin-treated groups and the value obtained after a 30 min stabilization period in the control groups. -Δ% shows the decrease. ***p<0.001 significantly different from the respective control.

**Figure 3:**
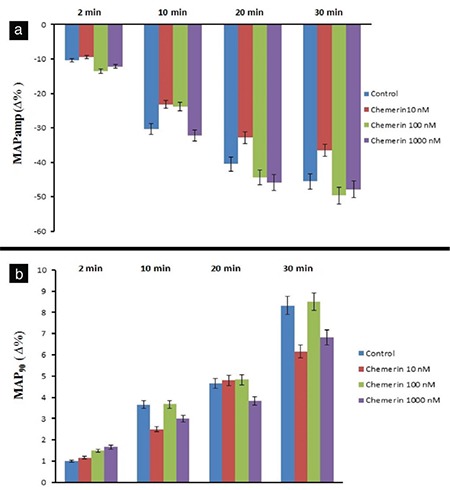
Time-dependent effect of chemerin on the MAPamp (a) and MAP_90_ (b). Δ% represents the change as a percentage of the 0 min value, that is, the value obtained prior to the administration of chemerin in chemerin groups and the value obtained after a 30 min stabilization period in the control groups. -Δ% and +Δ% show the decrease and increase, respectively. MAPamp: monophasic action potential amplitude; MAP_90_ duration at 90% repolarization

**Figure 4:**
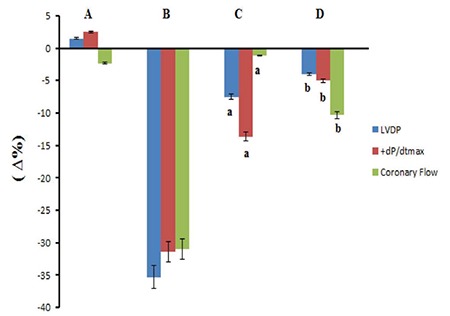
Effects of the application of 10 μM dose of L-NAME and 10 μM dose of L-NAME with 1000 nM chemerin on the LVDP, +dP/dt_max_, and coronary flow at 2 min (n=7). Δ% shows the decrease. a; p<0.001 significantly different than control group, b; p<0.001 significantly different than chemerin 1000 nM. A: Control, B: Chemerin 1000 nM, C: L-NAME 10 μM, D: L-NAME 10 μM + Chemerin1000 nM. LVDP: left ventricular developed pressure; L-NAME: N(W)-nitro-L-arginine methyl ester

The 10 nM dose of chemerin decreased LVDP at 30 min (p<0.05), whereas the 100 and 1000 nM doses of chemerin also decreased LVDP at 2, 10, 20, and 30 min (p<0.001, Figure 1a). On the other hand, 1000 nM dose of chemerin decreased LVDP (35.33 ∆% maximum) at 2 min (p<0.001, Figure 1a). As illustrated in Figure 1b, the 10 nM chemerin decreased +dP/dt_max_ value at 30 min (p<0.05). Chemerin at 100 and 1000 nM doses also decreased +dP/dt_max_ values at 2, 10, 20, and 30 min of observation period (p<0.001). The 1000 nM dose of chemerin reduced +dP/dt_max_ value (31.3 ∆% maximum) at 2 min of the observation period (p<0.001). Chemerin induced reductions in LVDP and +dP/dt_max_ values in a dose-dependent manner, and maximal declines in LVDP and +dP/dt_max_ were observed at 30 min (Figure 1a, 1b, respectively). A total of 1000 nM chemerin reduced coronary flow for 31 ∆% maximum at 2 min compared with the control group (p<0.001, Figure 2). In addition, none of the chemerin doses influenced the heart rate, MAPamp, and MAP90 values during the 30 min observation (Figure 2a, 3a, 3b, respectively). Only the 10 μM dose of L-NAME reduced LVDP, +dP/dt_max_, coronary flow with values of 7.5 ∆%, 13.66 ∆%, and 1.16 ∆%, respectively, at 2 min compared with the control group (p<0.001). However, only 1000 nM chemerin reduced LVDP, +dP/dt_max_, and coronary flow values by 35.33 ∆%, 31.33 ∆%, and 31 ∆% respectively. In the combination 10 μM dose of L-NAME and chemerin groups, the decrease in LVDP, +dP/dt_max_, and coronary flow values reached 4 ∆%, 5 ∆%, and 10.33 ∆%, respectively. The 10 μM dose of L-NAME, together with 1000 nM dose of chemerin, antagonized the decreasing effects of 1000 nM chemerin on LVDP, +dP/dt_max_, and coronary flow values at 2 min (p<0.001, Figure 4).

### Effect of chemerin on gene expressions and cAMP and cGMP production

**Figure 5:**
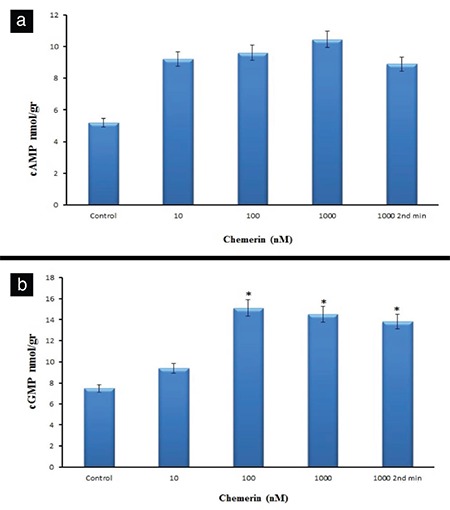
Effects of chemerin on the cAMP (a) and cGMP (b) amounts in the left ventricular tissue. *p<0.05 significantly different from the control, n=7. cAMP: cyclic adenosine monophosphate; cGMP: cyclic guanosine monophosphate

The heart tissue samples of the left ventricular were obtained after 30 min in all groups except at 1000 nM dose in the 2 min group, in which the samples were obtained at 2 min. The PI3Kα mRNA levels at all doses of chemerin are statistically insignificant. However, the PI3Kγ mRNA levels at 100 and 1000 nM of chemerin are statistically meaningful in comparison with the control group values. The expression of PI3Kγ mRNA was 1.8- and 1.63-fold lower under 100 and 1000 nM chemerin doses, respectively. Chemerin treatment caused no alteration in the *β1-AR* and *β2-AR* gene expressions. The 100 and 1000 nM chemerin doses significantly increased the eNOS mRNA levels. The results revealed that the increased eNOS mRNA gene expression in the 1000 nM dose chemerin group at 2 min is statistically more significant in comparison with those in the 100 and 1000 nM dose groups. The expression of eNOS gene increased by 1.91-fold for 100, 1.95-fold for 1000, and 2.16-fold for 1000 nM dose chemerin group at 2 min (Table 1). Furthermore, chemerin significantly decreased the mRNA levels of CaV1.2 gene expression (by 1.64, 1.69, and 1.4-fold for the 10, 100, and 1000 nM dose groups, respectively). Although the dose-dependent increases were observed in cAMP values measured in chemerin-treated hearts, these changes presented no statistical significance (Figure 5a). The treatment with 10 nM chemerin caused no substantial changes in the cGMP amount. However, the 100, 1000, and 1000 nM dose chemerin groups exhibited significantly increased the cGMP amounts at 2 min in comparison with the control group (p<0.001, Figure 5b).

## DISCUSSION

The present study showed that chemerin significantly reduced LVDP and +dP/dt_max_ values in isolated perfused rat hearts. This study also demonstrated that the treatment of isolated hearts with chemerin protein increased the eNOS mRNA and cGMP levels and reduced the expression of CaV1.2 gene in cardiac tissues. This research reported for the first time that acute chemerin treatment of isolated perfused rat leads to decreases in cardiac contractility. In addition, in this work, the negative inotropic effects of chemerin were antagonized with L-NAME. The findings revealed that NO may mediate the negative inotropic effects of chemerin.

Considering that adipocytokines activate several signaling pathways, chemerin could induce the NO synthesis through activation of the PI3K/Akt/eNOS pathways. The activation of the PI3K/Akt/eNOS signaling pathway results in the phosphorylation of Akt; phosphorylated Akt enhances the NO production (13). NO activates the soluble guanyl cyclase, subsequently leading to the production of cGMP. Subsequently, the cGMP-dependent protein kinase G (PKG) is activated. PKG reduces the amount of CaV1.2 mRNA and consequently reduces the amount of Ca^2+^ entering the heart muscle cells by decreasing the Ca^2+^ channel flow. PKG also exerts negative inotropic effects by diminishing the L-type Ca^2+^ channel currents and through the desensitization of cardiac myofilaments to Ca^2+^ (13,14). In this manner, the reduction of Ca^2+^ entering the cells results in a negative inotropic effect on the heart. Therefore, whether changes in the expression of genes are related to the PI3K/Akt/eNOS pathways in chemerin treatment isolated rat hearts must be investigated. To this end, we studied the expression of the genes of Cav1.2, PI3Kα, PI3Kγ, and eNOS on isolated rat hearts.

The application of chemerin to isolated rat hearts resulted in a statistically significant increase in the eNOS mRNA levels in comparison with the controls. In addition, with the increase in eNOS gene expression, the amount of cGMP in the cell also increased. NO contributes to the fine-tuning of the excitation-contraction coupling in the myocardium (15). The low concentrations of NO lead to positive inotropic effects, (16) whereas its high concentrations of NO result in the opposite trend. According to recent studies, the general idea regarding NO is that it causes biphasic effects on the myocardial function (15,16,17,18). These findings also support that NO mediates the negative inotropic effects of chemerin. NO is an important biological messenger which plays an important role in regulating cardiac functions, such as cardiac contraction, heart rate, and vascular tone. The disequilibrium of NO production is associated with cardiovascular disorders, including ischemic heart disease, hypertension, heart failure, coronary artery disorder, and arrhythmia (16). Studies have reported that endogenous eNOS activation is associated with ventricular arrhythmias in dogs. Massion ve ark. (15) observed that the amount of eNOS mRNA in myocytes decreased in the late phase of hypertrophic cardiomyopathy and heart failure. In the early period of ischemia and reperfusion, the amount of eNOS in the heart increases; the increase in NO protects against cardiac ischemia and reperfusion injury with various mechanisms (18). The cardiovascular system is complex given its own system and functions similar to those of the endothelial cells. In addition, the cardiovascular system plays an important role by interfering with other systems thorough various mechanisms. Research suggests that chemerin acts as a central actor in human physiology and pathology. For this reason, understanding chemerin, as a global protein in the cardiovascular system, will allow us to better understand the pathologies associated with chemerin and to apply appropriate treatments (3).

PI3Kα is a subunit of the PI3K signaling pathway, and the decrease in its signal reduces the number of L-type Ca^+2^ channels present in the heart cells; a decreased L-type Ca^+2^ channel current results in reduced contractility (19). In other words, PI3Kα participates in the positive regulation of heart contraction. *In vitro* contractility also increased due to the increased PI3Kα expression in the adult mice (20). In our study, we determined no statistically significant change in the PI3Kα gene expression levels in the chemerin groups in comparison with the control group. Considering the positive relationship between PI3Kα and the L-type Ca^+2^ channel current, we concluded that the significant decrease in the mRNA level of CaV1.2 gene in chemerin-treated groups is unrelated to PI3Kα.

PI3Kγ is another subunit of the PI3K signaling pathway. This subunit plays a role in regulating cardiac contraction. The catalytic subunit of PI3Kγ, p110γ, regulates the amount of cAMP (21). p110γ activates phosphodiesterases (PDEs); activated PDEs cause the destruction of cAMP. This condition negatively affects the contractility by reducing the amount of cAMP in the cell (21,22). In our study, statistically significant reductions in PI3Kγ gene expression levels were observed in the chemerin-treated groups. The literature shows that intracellular cAMP levels increase with decreasing PI3Kγ gene expression levels. The amounts of cAMP that is required to be evaluated together with the decreased PI3Kγ gene expression showed no statistically significant increase but demonstrated a 2-fold increase in comparison with the control values (23,24,25). The insignificant increase in the amount of cAMP and decrease in the PI3Kγ levels are consistent with the observed negative inotropic effects of chemerin.

The cardiac ARs in the myocardium contribute to the control of inotropy, rate of relaxation with the changing levels of intracellular Ca^2+^, and heart rate. Approximately 75% of the cardiac ARs are β1-AR, and the rest comprise β2-AR (26). In this research, the β1-AR and β2-AR gene expressions were studied to investigate whether changes in the expression of the genes are related to the contraction of isolated rat heart treated with chemerin. However, upon comparison of the chemerin-treated and control groups, no statistically significant difference was found in both gene expressions. In addition, no statistically significant difference in heartbeats was determined between the chemerin-administered and control groups. These findings suggest that chemerin could not mediate the effects of β1-AR and β2-AR on heart contractility. This compound also plays no part in the regulation of heart rate. As a result of the stimulation of β-ARs, the literature suggests that the level of cAMP in the cell increases; consequently, the contraction force of the heart increases (26). However, in our study, no significant increase was observed in the cAMP level. The statistically insignificant increase in the amount of cAMP in the chemerin-treated groups may be due to a decrease in the PI3Kγ gene expression level, leading to an increase in cAMP (25).

In our study, 2 min after the application of 1000 nM chemerin, the volume of coronary flow decreased by 30%-40%, and this decrease showed statistical significance. In addition, coronary flow volumes during the application of the chemerin remained below the control values. Lobato et al. (27) have demonstrated that chemerin enhanced the vasoconstriction responses of the α1-adrenergic agonist phenylephrine and endothelin-1 in rat aorta. Watts et al. (28) showed that the application of 1000 nM dose of chemerin caused contraction on the isolated thoracic aorta from obese and hypertensive rats and the isolated mesenteric arteries from obese humans. They also observed that the NO synthase inhibitor N-ω-nitro-L-arginine increased the contractile responses caused by chemerin. In our study, the reduction in the coronary flow may be attributed to the reduced heart contractility and the vasoconstrictor effect of chemerin on the 1000 nM dose group. The expectation was that eNOS would lead to a rise in coronary flow; however, the 10 and 100 nM chemerin doses caused no alterations on this variable. The coronary perfusion pressure is a good index of coronary vascular tone. However, in our study, the coronary effluent amounts could be measured from the collection of the coronary effluent after 1 min in a graduated cylinder instead of coronary perfusion pressure. Although the eNOS mRNA quantities increased, no increase was observed in the coronary flow. Thus, the unexpected results may be also the outcomes of the differences in the methods employed.

MAPamp depends on the intracellular diffusion of Na^+^ ions from Na^+^ channels in the first phase of MAP (phase 0) (29). In our study, chemerin caused no significant change in the values of MAPamp. This finding suggests that chemerin protein caused no alteration in the Na^+^ channel current during phase 0. On the other hand, MAP90 provides information about the repolarization phase of AP; it is inversely proportional to the number of heartbeats (30). The MAP90 showed a statistically insignificant change in the chemerin-treated groups compared with the control groups. The non-significant changes in the MAP90 in this study may be due to the insignificant change in the gene expression levels of β1-2-ARs and consequent negligible changes in heartbeats.

This study is the first attempt to demonstrate that chemerin exerts negative inotropic effects while decreasing the LVDP, +dP/dt_max_, and coronary flow. On the other hand, L-NAME totally reversed the negative inotropic effects of chemerin. We suggest that chemerin possesses a negative inotropic action, and NO may mediate this action. For the first time, we observed that chemerin reduces the PI3Kγ and CaV1.2 gene expressions but enhances the eNOS mRNA and tissue cGMP levels. This study argues that the negative inotropic effects generated by chemerin is associated with the increased amount of eNOS mRNA and decreased CaV1.2 mRNA levels. However, the relationship of negative inotropic effect induced by chemerin to cardiac and vascular pathology must be demonstrated *in vivo* and replicated in human tissues. Further and more advanced research is required to explore the potential impact of our findings on the pathology of cardiovascular illnesses and the potential role of chemerin in the treatment processes.

## Figures and Tables

**Table 1:**
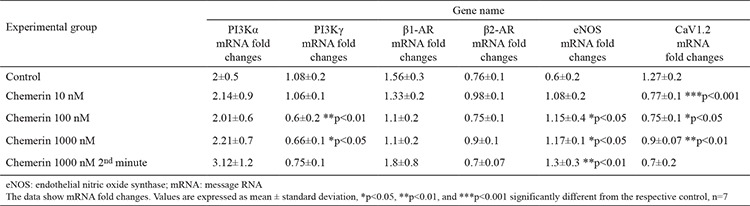
Relative gene expressions of PI3Kα, PI3Kγ, β1-AR, β2-AR, eNOS, and CaV1.2 in the control and chemerin-treated groups
